# TIGAR regulates DNA damage and repair through pentosephosphate pathway and Cdk5-ATM pathway

**DOI:** 10.1038/srep09853

**Published:** 2015-04-30

**Authors:** Hong-Pei Yu, Jia-Ming Xie, Bin Li, Yi-Hui Sun, Quan-Geng Gao, Zhi-Hui Ding, Hao-Rong Wu, Zheng-Hong Qin

**Affiliations:** 1 Department of General Surgery, the Second Affiliated Hospital of Soochow University, Suzhou 215004, China; 2 Department of Pharmacology and Laboratory of Aging and Nervous Diseases, Jiangsu Key Laboratory of Translational Research and Therapy for Neuro-Psycho-Diseases, College of Pharmaceutical Science, Soochow University Suzhou 215123, China; 3 Department of General Surgery, the First People’s Hospital of Wu Jiang, Suzhou 215004, China

## Abstract

Previous study revealed that the protective effect of TIGAR in cell survival is mediated through the increase in PPP (pentose phosphate pathway) flux. However, it remains unexplored if TIGAR plays an important role in DNA damage and repair. This study investigated the role of TIGAR in DNA damage response (DDR) induced by genotoxic drugs and hypoxia in tumor cells. Results showed that TIGAR was increased and relocated to the nucleus after epirubicin or hypoxia treatment in cancer cells. Knockdown of TIGAR exacerbated DNA damage and the effects were partly reversed by the supplementation of PPP products NADPH, ribose, or the ROS scavenger NAC. Further studies with pharmacological and genetic approaches revealed that TIGAR regulated the phosphorylation of ATM, a key protein in DDR, through Cdk5. The Cdk5-AMT signal pathway involved in regulation of DDR by TIGAR defines a new role of TIGAR in cancer cell survival and it suggests that TIGAR may be a therapeutic target for cancers.

A large number of studies found that exposure of cancer cells to acute hypoxia or genotoxic drugs induced a DDR (DNA damage response)^1^. The phosphopentose pathway (PPP), which converts glucose-6-phosphate to ribose-5-phosphate for synthesis of nucleotides and NADPH to reduce DNA damage caused by ROS was reported to be activated in DDR^2^. Spitz et al reported that the PPP was upregulated to produce more NADPH to reduce the hydroperoxide toxification for cancer cells^3^,^4^. Cosentino et al reported that G6PD (Glucose-6-phosphate dehydrogenase) activity, the rate-limiting enzyme of PPP is required for DNA repair process^5^.

Hepatocellular carcinoma (HCC), characterized by rapid recurrence and poor prognosis is the most common primary malignancy of the liver and the third-leading cause of cancer death^6^,^7^. Besides surgical resection or liver transplantation, transcatheter arterial chemoembolization (TACE) has been a beneficial treatment for unrespectable or relapsed HCC^8^,^9^,^10^. TACE induced a reduction in tumor size mainly due to ischemia resulting from embolization^11^ which expose tumor to acute hypoxia. Currently chemotherapeutic agents like doxorubicin, epirubicin, mitomycin and cisplatin were used for TACE^11^. Epirubicin was reported to block DNA replication either through direct DNA damage or indirectly through inhibition of replication proteins such as topoisomerase. Thus, one mechanism of epirubicin combined TACE treatment for HCC was through induction of synergistic DNA damage. DDR results in DNA repair, suppression of general translation, cell cycle arrest and, ultimately, either cell survival or cell death^12^. In caner therapy, DDR protects against genomic instability that enable cancer cells to become resistant to ionization radiation (IR) and chemotherapy by enhancing DNA repair of the lesions^13^,^14^.

TIGAR (TP53-induced glycolysis and apoptosis regulator) functions to lower fructose-2,6-bisphosphate (Fru-2,6-P2) levels and upregulate G6PD^15^ in cells, resulting in an inhibition of glycolysis and enhancement of the PPP to produce NADPH and ribose-5-phosphate, which are crucial for nucleotide synthesis and DNA repair^16^. Several recent studies have reported elevated TIGAR expression in human cancers such as glioblastoma^17^, invasive breast cancers^18^ and colorectal cancers (our unpublished observations). We have previously reported that TIGAR plays a pro-survival role in cancer cells through increase PPP flux^19^. Whether the elevated TIGAR expression protects DNA damage induced by chemotherapeutic agents or hypoxia has not been explored. Here, we present our findings on a novel role of TIGAR in DNA damage and repair.

## Results

### TIGAR knockdown increased DNA damage

**Figure 1:**
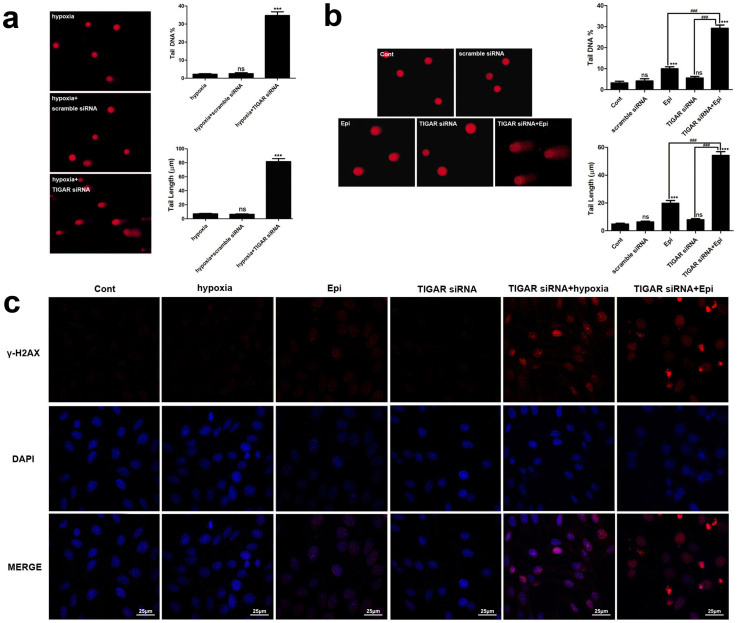
TIGAR knockdown increased DNA damage. Knockdown of TIGAR in HepG2 cells was achieved with transient transfection of TIGAR siRNA. Forty-eight h after transfection, HepG2 cells were then treated with 200 mM CoCl_2 _or 2.5 μg/ml epirubicin for 10 h and 12 h, respectively. (a) CoCl_2_-induced DNA damage in TIGAR knockdown HepG2 cells. Left: representative images of Comet assay. Right: quantification of Comet tail DNA% and tail length. (b) Epirubicin-induced DNA damage in TIGAR knockdown HepG2 cells. Left: representative images of Comet assay. Right: quantification of Comet tail DNA% and tail length. (c) Distribution of γ-H2AX in HepG2 cells treated with 200 nM CoCl_2 _or 2.5 μg/ml epirubicin after TIGAR knockdown. HepG2 cells were treated as described above and were analyzed with a confocal microscopy. γ-H2AX was stained red and the nucleus was stained blue. Scale bar = 25 μm. Values are means ± SD from 3 independent experiments. *** p < 0.001; ns, p > 0.05 versus control group; ### p < 0.001 versus corresponding groups.

To define a role of TIGAR in DNA damage, TIGAR expression was knocked down with siRNA in HepG2 cells. Epirubicin, a DNA damaging anticancer agent and CoCl_2_, which was used to imitate hypoxia condition, was applied to induce DNA damage in TIGAR knockdown HepG2 cells. DNA damage in HepG2 cells after TIGAR knockdown with or without treatment of epirubicin or CoCl_2 _was detected by Comet assay. Results showed that treatment with CoCl_2_ (200 μM) or epirubicin (2.5 μg/ml) for 10 or 12 h in control cells induced minor DNA damage. Knockdown of TIGAR significantly increased DNA damage after treatment with the same concentrations of CoCl_2_ or epirubicin as evidenced by increased percentage of tail DNA content and tail length of the Comet (Fig. 1a and b). A similar result was also observed in H1299 and HTC116 cells (Fig. S1a and b).

To further confirm DNA damage, the phosphorylation status of H2AX (γ-H2AX), a sensitive indicator of DNA double-strand breaks produced by DNA damage response was determined with immunoflurescence. Consistent with the Comet assay, in TIGAR knockdown HepG2 cells, CoCl_2_ or epirubicin robustly elevated the levels of γ-H2AX in the nucleus (Fig. 1c). These results demonstrated that TIGAR knockdown exacerbated DNA damage caused by CoCl_2_ or epirubicin. Furthermore, Brdu incorporation assay revealed that TIGAR knockdown also reduced DNA synthesis after epirubicin or CoCl_2 _treatment (Fig. S2).

### TIGAR knockdown increased DNA damage by suppressing PPP

**Figure 2:**
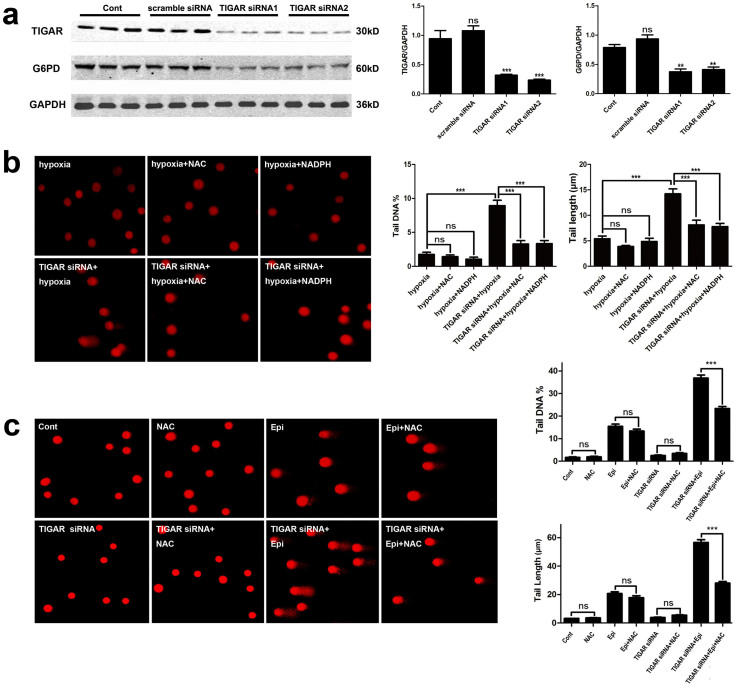
TIGAR knockdown increased DNA damage through increases in ROS. HepG2 cells or TIGAR knockdown HepG2 cells were all treated with CoCl_2 _(200 nM) for 10 h or epirubicin (2.5 μg/ml) for 12 h, and NAC, NADPH or ribose were added 2 h before the treatment of CoCl_2 _or epirubicin. DNA damage was detected with the Comet assay. (a) G6PD expression detected with Western blot analysis after TIGAR knockdown in HepG2 cells. GAPDH was used as a loading control. Quantitative analysis was performed with Image J. (b) DNA damage in TIGAR knockdown HepG2 cells treated with CoCl_2 _combined with or without treatment of NAC or NADPH. Left panel: representative images of Comet assay. Right panel: quantification of Comet tail DNA% and tail length. (c) DNA damage in TIGAR knockdown HepG2 cells treated with epirubicin combined with or without treatment of NAC. Left panel: representative images of Comet assay. Right panel: quantification of Comet tail DNA% and tail length. Values are means ± SD from 3 independent experiments. *** p < 0.001, ns p > 0.05 versus corresponding Groups.

**Figure 3:**
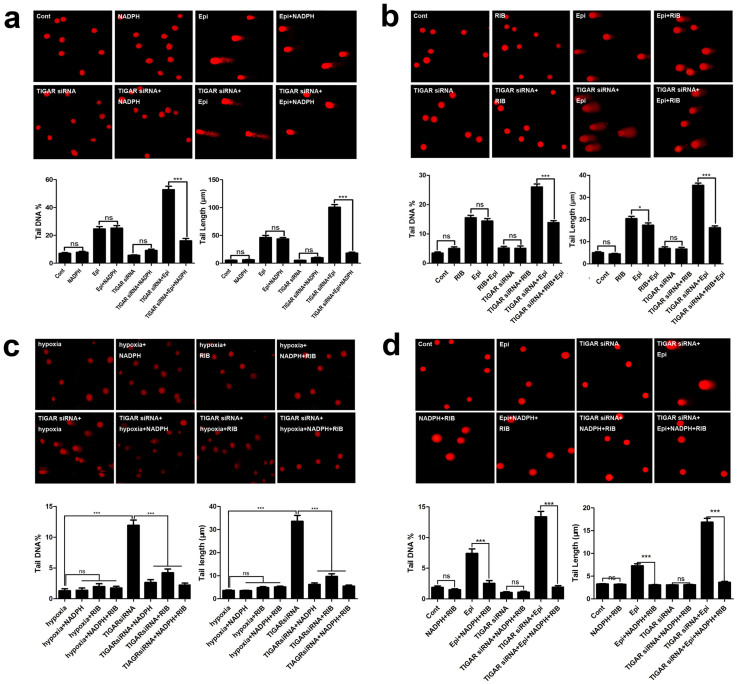
TIGAR knockdown increased DNA damage by reducing PPP. HepG2 cells or TIGAR knockdown HepG2 cells were treated with CoCl_2 _(200 nM) for 10 h or epirubicin (2.5 μg/ml) for 12 h, and NADPH or ribose was added 2 h before the treatment of CoCl_2 _or epirubicin. (a) DNA damage in TIGAR knockdown HepG2 cells or control cells treated with epirubicin combined with NADPH. Upper panel, representative images of Comet assay. Lower panel, quantification of Comet tail DNA% and tail length. (b) DNA damage in TIGAR knockdown HepG2 cells treated with epirubicin combined with or without ribose. Upper panel, representative images of Comet assay. Lower panel, quantification of Comet tail DNA% and tail length. (c) DNA damage in TIGAR knockdown HepG2 cells or control cells treated with CoCl_2_ combined with NADPH, ribose alone or both. Upper panel, representative images Comet assay. Lower panel, quantification of comet tail DNA% and tail length. (d) DNA damage in TIGAR knockdown HepG2 cells or control cells treated with epirubicin combined with NADPH and ribose. Upper panel, representative images of Comet assay. Lower panel, quantification of Comet tail DNA% and tail length . Values are means ± SD from 3 independent experiments. *** p < 0.001, ns p > 0.05 versus control group.

TIGAR was reported to activate PPP to produce ribose-5-phophate, a nucleotide precursor, and NADPH to reduce ROS levels^16^. As a previous study reported that G6PD was also regulated by TIGAR^15^, this study investigated if G6PD expression was down regulated by knockdown of TIGAR. The results showed a decreased level of G6PD after TIGAR knockdown (Fig. 2a). Our previous study had demonstrated that knockdown of TIGAR reduced PPP flux^19^. To investigate if the increased DNA damage by TIGAR knockdown after treatment of epirubicin or CoCl_2_ was due to reduced supply of PPP products and the elevated ROS levels, NADPH, ribose and the ROS scavenger NAC were supplemented. In HepG2 cells, epirubicin (2.5 μg/ml) or CoCl_2 _(200 μM) induced DNA damage was enhanced by knockdown of TIGAR. In this treatment regime, supplementation of NAC (10 mM) decreased the percentage of tail DNA content and tail length of the Comet (Fig. 2b and c), suggesting that an increase in ROS after TIGAR knockdown is partially responsible for exacerbation of DNA damage. Similarly, NADPH (10 μM) or ribose (10 mM) was added to cell culture medium when TIGAR knockdown HepG2 cells were treated with 2.5 μg/ml epirubicin or 200 μM CoCl_2_. Comet assay showed that the percentage of tail DNA content and tail length of the Comet were decreased after applying exogenous NADPH or ribose (Fig. 2b; Fig. 3a–c). Furthermore, supplying NADPH and ribose simultaneously resulted in a greater abolishment of DNA damage enhancement by TIGAR knockdown (Fig. 3c and d). These results revealed that knockdown of TIGAR increased DNA damage in HepG2 cells after epirubicin or CoCl_2 _treatment through inhibiting PPP.

### Nuclear translocation of TIGAR under genome stress or hypoxia condition

**Figure 4:**
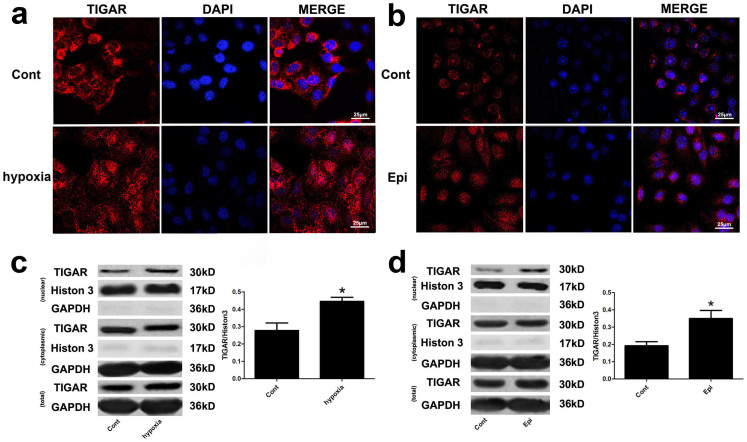
The nuclear translocation of TIGAR under genome stress or hypoxia condition. (a) The nucleus translocation of TIGAR after treatment of HepG2 cells with 200 mM CoCl_2 _for 8 h. Fluorescence intensity of TIGAR in the nucleus was detected with a confocal microscopy. TIGAR was stained red and the nucleus was stained blue. Scale bar = 25 μm. (b) The nuclear translocation of TIGAR after treatment of 2.5 μg/ml epirubicin for 12 h. Fluorescence intensity of TIGAR in the nucleus was detected with a confocal microscopy. TIGAR was stained red and the nucleus was stained blue. Scale bar = 25 μm. (c) The nuclear TIGAR protein after treatment with CoCl_2_. The nuclear proteins were extracted and TIGAR protein level in the nucleus and cytosol was detected with Western blot analysis. Histon-H3 was used as a loading control for the nuclear protein and GAPDH was used as a loading control for cytosolic proteins. Quantitative analysis was performed with Image J. (d) The nuclear TIGAR protein after treatment with epirubicin. The nuclear proteins were extracted and TIGAR protein level in the nucleus and cytosl was detected with Western blot analysis. Histon-H3 was used as a loading control for the nuclear proteins and GAPDH was used as a loading control for the cytosolic proteins. Quantitative analysis was performed with Image J. Values are means ± SD from 3 independent experiments. * p < 0.05 versus control group.

Zhang H et al. reported that the nuclear translocation of thioredoxin-1 (TRX1), a redox-sensitive oxidoreductase that plays a critical role in DNA damage in irradiated cells was regulated by TIGAR^20^. Consistently, our current results showed that under genome stress or in hypoxia condition, TRX1 was translocated to the nuclei and its translocation was regulated by TIGAR in HepG2 cells (Fig. S3). More importantly, the present study found an increased nuclear localization of TIGAR after HepG2 cells were treated with CoCl_2_ or epirubicin. Immunofluorescence detection of TIGAR protein showed a significant increase in TIGAR immunoreactivity in the nuclei after treatment with 200 μM CoCl_2 _for 8 h (Fig. 4a) or 2.5 μg/ml epirubicin for 12 h (Fig. 4b). The increase in nuclear localization of TIGAR was further confirmed by cell fractionations. Western blot analysis showed an increase in TIGAR protein level in the nuclear fraction after HepG2 cells treated with CoCl_2 _(Fig. 4c) or epirubicin (Fig. 4d). A similar increase in nuclear TIGAR was also observed in SMMC7721 cells (Fig. S4).

### TIGAR regulated Cdk5-ATM pathway

**Figure 5:**
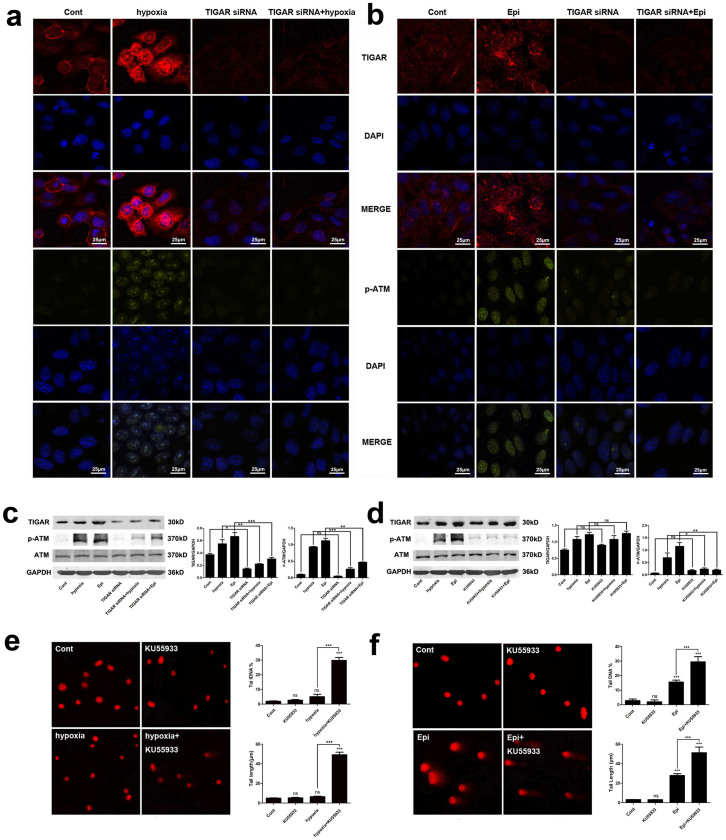
TIGAR protected DNA from damage through phosphorylating ATM. (a and b) HepG2 cells or TIGAR knockdown HepG2 cells were treated with 200 mM CoCl_2 _or 2.5 μg/ml epirubicin. Fluorescence intensity of phosphorylated ATM protein was detected with a confocal microscopy. P-ATM was stained red and the nucleus was stained blue. Scale bar = 25 μm. (c) Cells were treated as described above. Protein levels of TIGAR, phosphorylated ATM or total ATM was detected with Western blot analysis. GAPDH was used as a loading control. Quantitative analysis was performed with Image J. (d) Cells were treated with 200 mM CoCl_2 _or 2.5 μg/ml epirubicin and ATM inhibitor KU55933 was added in corresponding group. Expression of TIGAR, phosphorylated ATM and total ATM protein was detected with Western blot analysis. GAPDH was used as a loading control. Quantitative analysis was performed with Image J. (e) DNA damage after ATM was inhibited by KU55933 under hypoxia condition. HepG2 cells were treated with or without 200 mM CoCl_2 _for 10 h, and KU55933 was added 1 h before CoCl_2 _treatment in corresponding group. Left, representative images of Comet assay. Right, quantification of Comet tail DNA% and tail length. (f) DNA damage after ATM was inhibited by KU55933 after epirubicin treatment. HepG2 cells were treated with or without 2.5 μg/ml epirubicin for 12 h, and KU55933 was added 1 h before epirubicin treatment in corresponding group. Left, representative images of Comet assay. Right, quantification of Comet tail DNA% and tail length. Values are means ± SD from 3 independent experiments. *p <0.05, **p < 0.01, *** p < 0.001, ns p > 0.05 versus corresponding groups.

To determine a role of TIGAR in DDR, Ataxia-telangiectasia mutated (ATM), a protein that plays a major role in initiating the DDR was examined. It was found that both TIGAR and ATM were induced by epirubicin or CoCl_2_ in HepG2 cells (Fig. 5a and b) and in SMMC7721 cells (Fig. S5). To determine the role of TIGAR in ATM activation, Western blot analysis of TIGAR, phosphorylated and total ATM protein were performed, and the results showed that the induction of TIGAR and p-ATM was temporally correlated (Fig. 5c). Furthermore, knockdown of TIGAR (Fig. 5c) or treatment with the ATM specific inhibitor KU55933 inhibited phosphorylation of ATM, while ATM KU55933 did not affect the expression of TIGAR (Fig. 5d). To determine the role of ATM in CoCl_2_- or epirubicin-induced DDR, HeG2 cells were pre-treated with the ATM specific inhibitor KU55933 and the Comet assay was performed. After treatment with KU55933, DNA damage was markedly increased after treatment of 200 mM CoCl_2 _(Fig. 5e) or 2.5 μg/ml epirubicin (Fig. 5f). These results suggested that TIGAR regulated ATM phosphorylation and phosphorylated ATM was involved in DDR.

**Figure 6:**
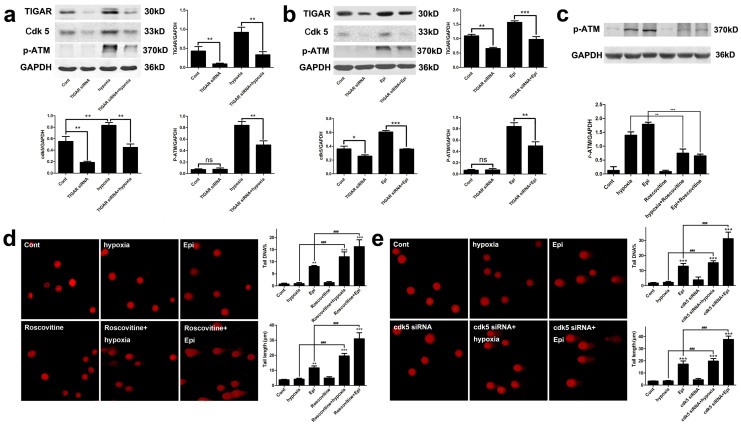
TIGAR protected DNA from damage through regulating Ckd5. (a) Expression of Cdk5 and p-ATM protein after TIGAR knockdown in hypoxia condition. HepG2 cells or TIGAR knockdown HepG2 cells were treated with 200 mM CoCl_2_ and protein levels of Cdk5 and p-ATM were detected with Western blot analysis. GAPDH was used as a loading control. Quantitative analysis was performed with Image J. (b) Expression of Cdk5 and p-ATM protein after TIGAR knockdown cells were treated with epirubicin. HepG2 cells or TIGAR knockdown HepG2 cells were treated with 2.5 μg/ml epirubicin and protein levels of Cdk5 and p-ATM were detected with Western blot analysis. GAPDH was used as a loading control. Quantitative analysis was performed with Image J. (c) The Cdk5 inhibitor roscovotine inhibited ATM phosphorylation. HepG2 cells were treated with 200 mM CoCl2 or 2.5 μg/ml epirubicin and roscovotine was added 24 h before. Protein levels of phosphorylated ATM were detected with Western blot analysis. GAPDH was used as a loading control. Quantitative analysis was performed with Image J. (d) DNA damage of HepG2 cells after treatment with 200 mM CoCl_2 _or 2.5 μg/ml epirubicin when Cdk5 was inhibited by roscovotine. Left panel, representative images of Comet assay. Right panel, quantification of Comet tail DNA% and tail length. Values are means ± SD from 3 independent experiments. * p < 0.05, **p < 0.01, *** p < 0.001, ns p > 0.05 versus control group; ### p < 0.001 versus corresponding groups. (e) DNA damage of HepG2 cells after treatment with 200 mM CoCl_2 _or 2.5 μg/ml epirubicin when Cdk5 was knocked down by Cdk5 siRNA. Left panel, representative images of Comet assay. Right panel, quantification of Comet tail DNA% and tail length. Values are means ± SD from 3 independent experiments. *** p < 0.001 versus control group; ### p < 0.001 versus corresponding groups.

Previous studies have reported that phosphorylation of ATM by Cdk5 mediates DDR signaling^18^. The present study showed that the expression of Cdk5 was upregulated in HepG2 cells by epirubicin or CoCl_2 _and its induction was robustly inhibited by knockdown of TIGAR (Fig. 6a and b). To determine a role of Cdk5 in phosphorylation of ATM and DNA damage, the Cdk5 inhibitor roscovotine was applied and p-ATM proteins were determined in the present study. The treatment with roscovotine markedly blocked CoCl_2_- or epirubicin-induced elevation in the level of p-ATM (Fig. 6c). Moreover, roscovotine significantly increased the CoCl_2_- or epirubocin-induced DNA damage (Fig. 6d). Furthermore, we knocked down Cdk5 with Cdk5 siRNAs and the effects of TIGAR on DDR after Cdk5 knockdown was determined with Comet assay. The results showed that knockdown of Cdk5 robustly enhanced DNA damage induced by CoCl_2_ or epirubocin (Fig. 6e). These results indicated that Cdk5 contributed to phosphorylation of ATM and TIGAR regulates expression of Cdk5.

## Discussion

DNA damage-inducing therapies targeting the rapidly dividing cancer cells with genotoxic agents have demonstrated clinical utility, however, it has become apparent that the DDR tempers the efficacy of these therapies^12^. The DDR rapidly recognizes DNA lesions and activates appropriate DNA repair mechanisms to maintain genome integrity^2^, which provides a common strategy for cancer-therapy resistance. DDR inhibition might enhance the effectiveness of radiotherapy and DNA-damaging chemotherapy. Various DDR-inhibitory drugs are in pre-clinical and clinical development^2^,^32^,^33^. ROS was reported to activate DDR^21^,^22^,^23^. TIGAR functions to inhibit glycolysis, resulting in higher intracellular NADPH and lower ROS. Thus the role and mechanisms by which TIGAR affect DDR are warranted to be studied.

Consistent with previous studies, TIGAR expression was elevated after treatment with epirubicin or CoCl2. Since TIGAR was able to enhance PPP, and PPP pathway was reportedly to be involved in DDR^16^,^5^, we thus explored the role of elevated TIAGR in DDR. The present results showed more severe DNA damage after TIGAR knockdown combined with epirubicin or CoCl2 treatment, suggesting a protective role of TIGAR on DNA damage. TIGAR expression was also increased in H1299, a TP53-deficient cell line treated with epirubicin. Knockdown of TIGAR in H1299 cells also aggravated DNA damage by epirubicin or Cocl2. The results suggested that the regulation of DDR by TIGAR could be independent of TP53 in H1299.

Reactive oxygen species (ROS) can induce a wide array of DNA damage including base oxidation, sugar fragmentation and single strand DNA breaks^12^. TIGAR decreases intracellular ROS levels through increasing NADPH generation^16^, and knockdown of TIGAR significantly increased ROS levels^16^. The present study demonstrated that the increased DNA damage after TIGAR knockdown was due to the elevated ROS levels, as the anti-oxidant NAC reduced DNA damage. Similar effects were obtained with supplementation of NADPH. However, compared to control cells, DNA damage was still higher, suggesting additional mechanisms were involved. The possibility is that ribose-5-phosphate generated by PPP may also play a role in DNA damage. It is expected that increased production of ribose-5-phosphate through PPP would promote the synthesis of nucleotides and repair of DNA lesions^5^. To evaluate if the increased DNA damage after TIGAR knockdown was also due to the reduced supply of ribose-5-phosphate. Ribose was added to HepG2 cells and Comet assay revealed a partial reduction of epirubicin- and CoCl_2_-induced DNA damage. DNA damage induced by epirubicin or CoCl_2 _treatment in TIGAR knockdown cells was almost restored to that of control cells after applying both NADPH and ribose simultaneously, suggesting that TIGAR protects from DNA damage through increasing supply of NADPH and ribose-5-phosphate. TIGAR knockdown also reduced the expression of G6PD, which was consistent with the study from Costanzo reporting that G6PD activity, a rate limiting enzyme of PPP, is required for DNA repair^5^.

More importantly, our study found that TIGAR protein translocated to the nucleus after HepG2 cells were treated with epirubicin or CoCl_2_. This raised a question if TIGAR can regulate nuclear proteins involved in DDR. Ataxia-telangiectasia mutated (ATM), a member of the phosphatidylinositol 3-kinase-related kinase (PIKK) families, plays a central role in initiating the DDR. ATM remains a homodimer while inactive, but undergoes trans-autophosphorylation at serine 1981 upon activation, leading to disassociation of the dimer, and allowing monomeric ATM to be recruited to dsDNA via an interaction with the MRE11-RAD50-NBS1 (MRN) complex^5^,^24^. Cosentino et al reported that ATM activates the PPP thus it promotes anti-oxidant defense and DNA repair^5^. We speculated that TIGAR might play a role in DDR through ATM. The current results showed that the phosphorylation of ATM increased in response to a genotoxic drug or hypoxia treatment. TIGAR knockdown reduced phosphorylation of ATM, however, expression of TIGAR was not affected when ATM was inhibited by ATM specific inhibitor, which indicates that TIGAR regulates DDR through phosphorylation of ATM. How does TIGAR affect the phosphorylation of ATM? Other investigators found that a number of DDR components can be phosphorylated by CDKs, and these modifications regulate checkpoint signaling and repair pathway choices^20^. Cdk5 can be activated by DNA damage^25^,^26^,^27^,^20^. The activation of Cdk5 by DNA damage directly phosphorylates ATM at serine 794, which is required for ATM autophosphorylation at serine 1981 to activate ATM kinase activity^27^. The present study found that epirubicin and CoCl_2_ upregulated Cdk5 along with TIGAR. To evaluate if phosphorylation of ATM by TIGAR was mediated through Cdk5, TIGAR was knocked down and the expression of Cdk5 was determined. The results showed that knockdown of TIGAR reduced the levels of Cdk5 as well as phosphorylated ATM. As Cdk5 plays an important role in the processes of DNA repair^28^,^29^, the present study utilized seliciclib (roscovitine) to define if Cdk5 was involved in doxrobicin- and CoCl_2_-induced phosphorylation of AMT. Roscovitine is consider a Cdk5 inhibitor and is currently in phase II clinical trial for cancer treatment^30^,^31^. Consistent with previous studies, our results showed a reduction in ATM phosphorylation when Cdk5 was inhibited by roscovitine, meanwhile, the epirubicin- or CoCl_2_-induced DNA damage was increased. To further test the involvement of Cdk5 in DDR regulation by TIGRA, Cdk5 was knocked down with siRNA. The results showed knockdown of Cdk5 enhanced epirubicin- and CoCl_2_-induced DNA damage. These studies indicated that TIGAR promotes DNA repair through activating Cdk5-ATM pathway.

**Figure 7:**
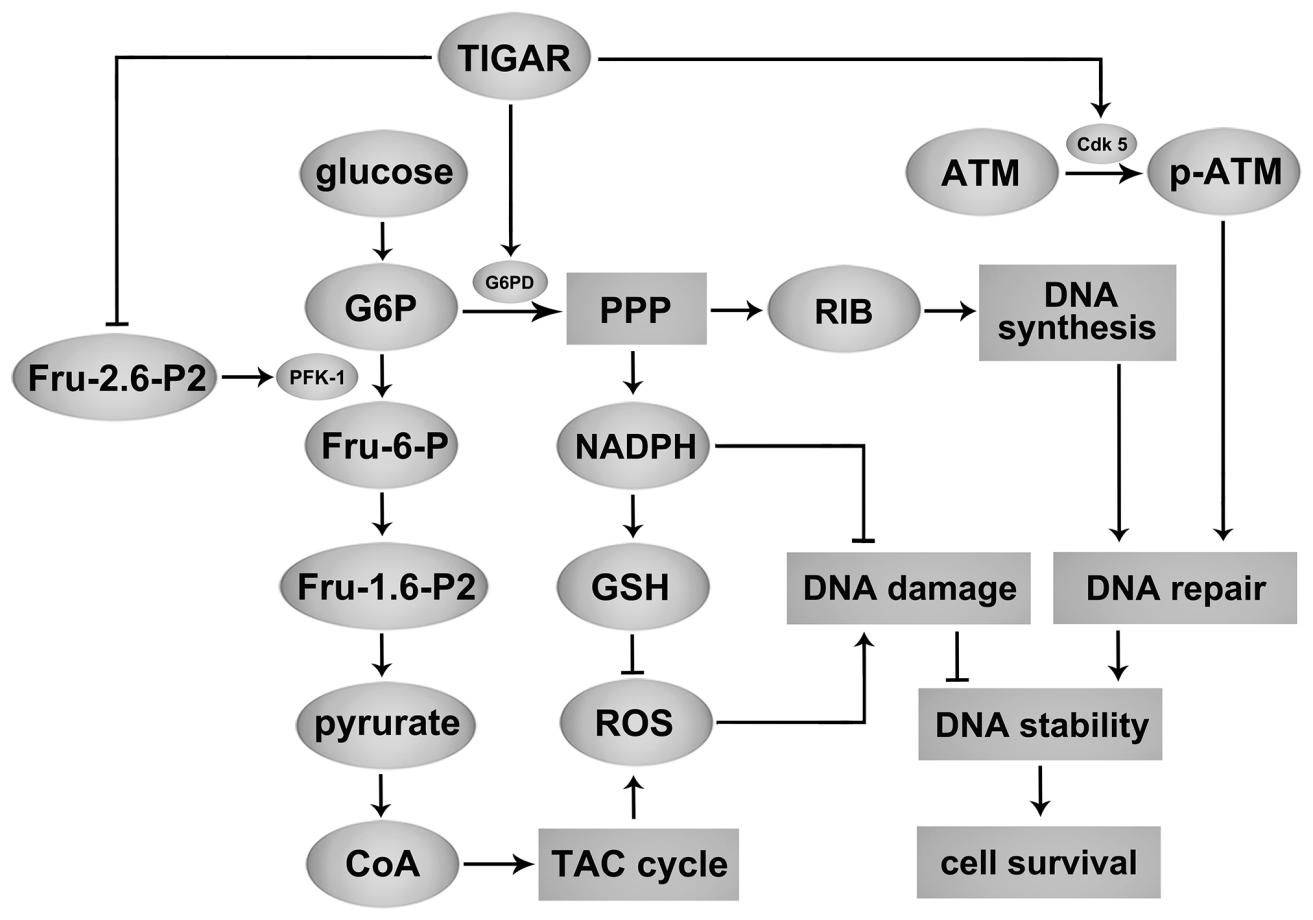
Proposed mechanisms by which TIGAR regulates DNA damage. TIGAR functions to lower Fru-2,6-P2 levels and upregulate G6PD in cells, resulting in an inhibition of glycolysis and enhancement of the PPP to produce NADPH and ribose-5-phosphate. TIGAR reduces DNA damage and enhances DNA repair through increasing generation of NADPH which decreases intracellular ROS levels and ribose which provides materials for synthesis of nucleotides. TIGAR also promotes DNA repair by increasing phosphorylation of ATM through Cdk5. Thus TIGAR favors cancer cell survival by increasing stability of DNA.

In summary, our data indicate that TIGAR reduces anti-cancer drug- and hypoxia-induced DNA damage and enhances DNA repair through increasing generation of NADPH and ribose. The nuclear Cdk5-ATM signaling pathway is involved in regulation of DNA repair by TIGAR (Fig. 7). These data suggest that TIGAR-regulated PPP may be a potential target for cancer therapy especially for those radiation and chemotherapy resistant cancers.

## Methods

### Cell culture

Human hepatocellular carcinoma derived HepG2 and SMMC7721 cells, Non-small-cell carcinoma derived NCI-H1299 cells and human colon carcinoma derived HCT116 cells were obtained from the American Type Culture Collection and cultured with Dulbecco’s modified Eagle medium (DMEM; Gibco, 11965500) supplemented with 10% fetal bovine serum (FBS; Wisten Inc, 086150008; Arizona, USA), 100 IU/ml penicillin and 100 IU/ml streptomycin in a humidified incubator at 37°C under 5% CO2 atmosphere, and passaged at pre-confluent densities by use of 0.25% trypsin solution every 2–3 days. Cells were stored and used within 3 months after resuscitation of frozen aliquots. All cells were used in accordance with the institutional guidelines and the study protocol was approved by the ethical committee of Soochow University.

### Transfection and RNA interference

To inhibit TIGAR or Ckd5 expression, small-interference RNA (siRNA) matching region 565–583 in exon 6 (TTAGCAGCCAGTGTCTTAG; TIGAR siRNA2) of the human TIGAR cDNA sequence was synthesized by GenePharma (Shanghai, China), and a scramble sequence (TTACCGAGACCGTACGTAT) was synthesized as a negative control. Cdk5 siRNA(h) was synthesized by Santa Cruz Biotechnology (cdk5 siRNA: sc-29263, Santa Cruz Biotechnology). Transient transfection was performed using the Lipofectamine 2000 reagent (Invitrogen, 11668019; California, USA) according to the manufacture’s protocol. HepG2 cells were plated at a density of 5 × 10^5^ cells in 6-well plates and were then transfected with TIGAR siRNAs using Lipofectamine 2000 reagent diluted in Opti-MEM Reduced Serum Medium 24 h later. The final concentration of TIGAR siRNA was 80 nM. Complete medium free of antibiotics was added to each well 6 h after transfection. Cells were trypsinized and harvested for Western blot analysis at the indicated times.

### Western blot analysis

Protein was extracted from cells using cell lysis solution with protease inhibitors (Roche, 04693159001; Basle, Switzerland) and phosphorylase inhibitors (Roche, 04906845001). Protein concentration was determined with a BCA protein assay kit (Thermo Fisher Scientific, 23227, Massachusetts, USA). Equal amounts of protein were fractionated on Tris-glycine SDS-polyacrylamide gels and subjected to electrophoresis and transferred to NC membranes. Membranes were blocked with TBS containing 5% (w/v) dry milk with 0.1% Tween 20, washed with TBS containing 0.1% Tween 20 (TBST), and then incubated overnight at 4°C with specific antibodies against TIGAR (1:1,000; Abcam, ab37910; Cambridge, UK), G6PD (1:1000; CST,#8866; Massachusetts, USA), Ckd5 (1:1000; Abcam, ab40773), p-ATM (1:1000; Abcam, ab81292), ATM (1:1000; Abcam, ab78), GAPDH (1:2000; Sigma, SAB1405848) in non-fat milk containing 0.1% NaN3. After washing in TBST, membranes were incubated with fluorescent secondary antibodies (1:10,000; Jackon ImmunoResearch, anti-rabbit, 711-035-152, anti-mouse, 715-035-150; West Grove, PA, USA) at room temperature for 1 h. Immunoreactivity was detected using ODYSSEY INFRARED IMAGER (Li-COR Biosciences, Nebraska, USA). The signal intensity of primary antibody binding was quantitatively analyzed with Image J software (W.S. Rasband, Image J, NIH, Bethesda, MD).

### Immunofluorescence

The HepG2 cells were seeded onto cover glass (Thermo Fisher Scientific, #032910-9) in 24 well plates. Thereafter, cells were washed with phosphate-buffered saline (PBS) for 5 minutes × 3 times. Then cells were treated with pre-cooled alcohol for 15 min. Cells were blocked in PBS, containing 1% BSA and 0.1% Triton X-100 for 1 hour at room temperature. Then the cells were incubated with primary antibody overnight at 4°C. After washing cells with PBS for 10 min × 3 times, the cells were incubated with Cy3-conjugated donkey anti-rabbit IgG (1:1000; Jackson ImmunoResearch Laboratories) for 1 h at room temperature. After 10 min × 3 times of washing with PBS, cells were incubated with DAPI for 10 min, the cells were dehydrated in increasing grades of ethanol and cover-slipped with Fluoromount Aqueous Mounting Medium (Sigma, F4680; Sant Louis, MO, USA). The slices were analyzed with a laser scanning confocal unit (Zeiss LSM 710, Carl Zeiss, Jena,Germany).

### Subcellular fractionation

Nuclear and cytosolic extracts were prepared according to the manufacturer’s instruction (Beyotime, Haimen, China). Briefly, cells were mixed with the cytoplasmic extraction buffer A on ice for 10 min. Suspension was shaken vigorously for 5 seconds and centrifuged at 16,000 *g* for 5 min at 4°C. The supernatant was collected as cytosolic extracts. The pellet was re-suspended in nuclear extraction buffer B on ice for 30 min. The resulting supernatant was used as nucleic fractions following centrifuge at 16,000 *g* for 10 min. All subcellular fractions were stored at −80°C.

### Comet assay

Cells were seeded onto 6-well plates at a density of 5 × 10^5^ cells for 24 h. Cells were then transfected with or without TIGAR siRNAs for 48 h, epirubicin or Cocl_2 _were added 12 h or 10 h, respectively, before the end of transfection. KU55933 was added 1 h before CoCl_2_ or epirubicin treatment. Roscovotine was applied 24 h before CoCl_2_ or epirubicin treatment. Cells were washed with PBS and trypsinized with 0.25% trypsin and then mixed with 0.6% low-melting-temperature agarose at 37°C. The microscopy slide was covered with a thin layer of 0.8% normal-melting agarose and solidified at 4°C for 10 min. The low-melting-temperature agarose mixed with cells was placed on the top of agarose covered microscopy slide. After solidifying for 10 min, the slides were immersed in a lysing solution for 2 h to lyse the cells and to permit DNA unfolding, then were placed on a horizontal gel electrophoresis unit that was filled with fresh electrophoretic buffer and electrophoresed at 25 V for 20 min. At the end of electrophoresis, the slides were washed with 0.4 M Tris (pH 7.5) to remove alkali and detergents and were then stained with ethidium bromide. The slides were observed with a fluorescent microscopy. Comet tail length and DNA% in the tail was measured with Comet Assay IV software (Perceptive Instruments Ltd., Suffolk,UK).

### Brdu incorporation assay

HepG2 cells were seeded onto cover glass in 24 well plates for 24 h and then transfected with or without TIGAR siRNA for 48 h, epirubicin or Cocl2 was added 12 or 10 h, respectively, before the end of experiment. Brdu (Sigma, B5002) was added to cell culture medium at a concentration of 10 μM 1 h before the end of experiment. After washed with PBS for 5 min × 3 times, cells were fixed with 95% ethanol for 15 min, and incubated with 0.1%Triton-X 100 for 15 min, and cells’ DNA was denatured with 4N HCl for 2 h. Cells were incubated with the primary anti-BrdU antibody (Abcam, ab8152) overnight at 4°C, followed by incubation with the Cy3-conjugated donkey anti-mouse IgG (1:800; Jackson ImmunoResearch Laboratories) for 1 h at room temperature. After washing with PBS, cells were incubated with DAPI for 10 min. Cells were cover-slipped with Fluoromount Aqueous Mounting Medium (Sigma, F4680). The slices were analyzed with a laser scanning confocal unit (Zeiss LSM 710, Carl Zeiss, Jena, Germany).

### Statistical analysis

All data were presented as means ± SD. Data were subjected to one-way ANOVA using the GraphPad Prism software statistical package (GraphPad Software, San Diego, CA, USA). When a significant group effect was found, post hoc comparisons were performed using the Newman–Keuls t-test to examine special group differences. Independent group t-tests were used for comparing two groups. Significant differences at p < 0.05, 0.01 and 0.001 are indicated by *, **, ***, respectively. All calculations were performed using the 14.0 SPSS software package (SPSS Inc.).

## Author Contributions

All listed authors contributed to the idea generation, design, and completion of this work. H.P.Y., J.M.X. contributed equally to the idea generation, experimental work and manuscript preparation. B.L., Y.H.S., Q.G.G. and Z.H.D. contributed to the experimental work and manuscript preparation. H.R.W. and Z.H.Q. guided the idea generation, experimental work and manuscript preparation. All authors reviewed the manuscript.

## Supplementary Material

Supplementary Information Supplementary information
