# Supplementary material for: TIGAR regulates DNA damage and repair through pentosephosphate pathway and Cdk5-ATM pathway
Source: Sci Rep. 2015 Apr 30;5:9853. doi: 10.1038/srep09853 (PMC4415581; doi:10.1038/srep09853)
Supplement: Supplementary Information [file srep09853-s1.pdf]

TIGAR regulates DNA damage and repair through pentosephosphate pathway and Cdk5-ATM pathway

Running title: TIGAR regulates DNA damage response

Hong-Pei Yu[1,*], Jia-Ming Xie[1,*], Bin Li[3,], Yi-Hui Sun[1], Quan-Geng Gao[3], Zhi-Hui Ding[2], Hao-Rong Wu[1,#], Zheng-Hong Qin[2,#]

[1]Department of General Surgery, the Second Affiliated Hospital of Soochow University, Suzhou 215004, China.

[2]Department of Pharmacology and Laboratory of Aging and Nervous Diseases, Jiangsu Key Laboratory of Translational Research and Therapy for Neuro-Psycho-Diseases, College of Pharmaceutical Science, Soochow University Suzhou 215123, China

[3]Department of General Surgery, the First People's Hospital of Wu Jiang, Suzhou 215004, China.

*: These authors contribute to this work equally.

#: Corresponding authors:

Hao-Rong Wu, MD

Department of General Surgery

The Second Affiliated Hospital of Soochow University

1055 San Xiang Road, Suzhou, 215006, China

Phone: 86-512-67783308

Fax: 86-512-67783308

Email: wuhaorong@vip.sina.com

Zheng-Hong Qin, PhD

Department of Pharmacology and Laboratory of Aging and Nervous Diseases

Soochow University School of Pharmaceutical Sciences

199 Ren Ai Road, Suzhou 215123, China

Phone: 86-512-65882071

Fax: 86-512-65882071

Email: qinzhenhong@suda.edu.cn

The authors disclose no potential conflicts of interest.

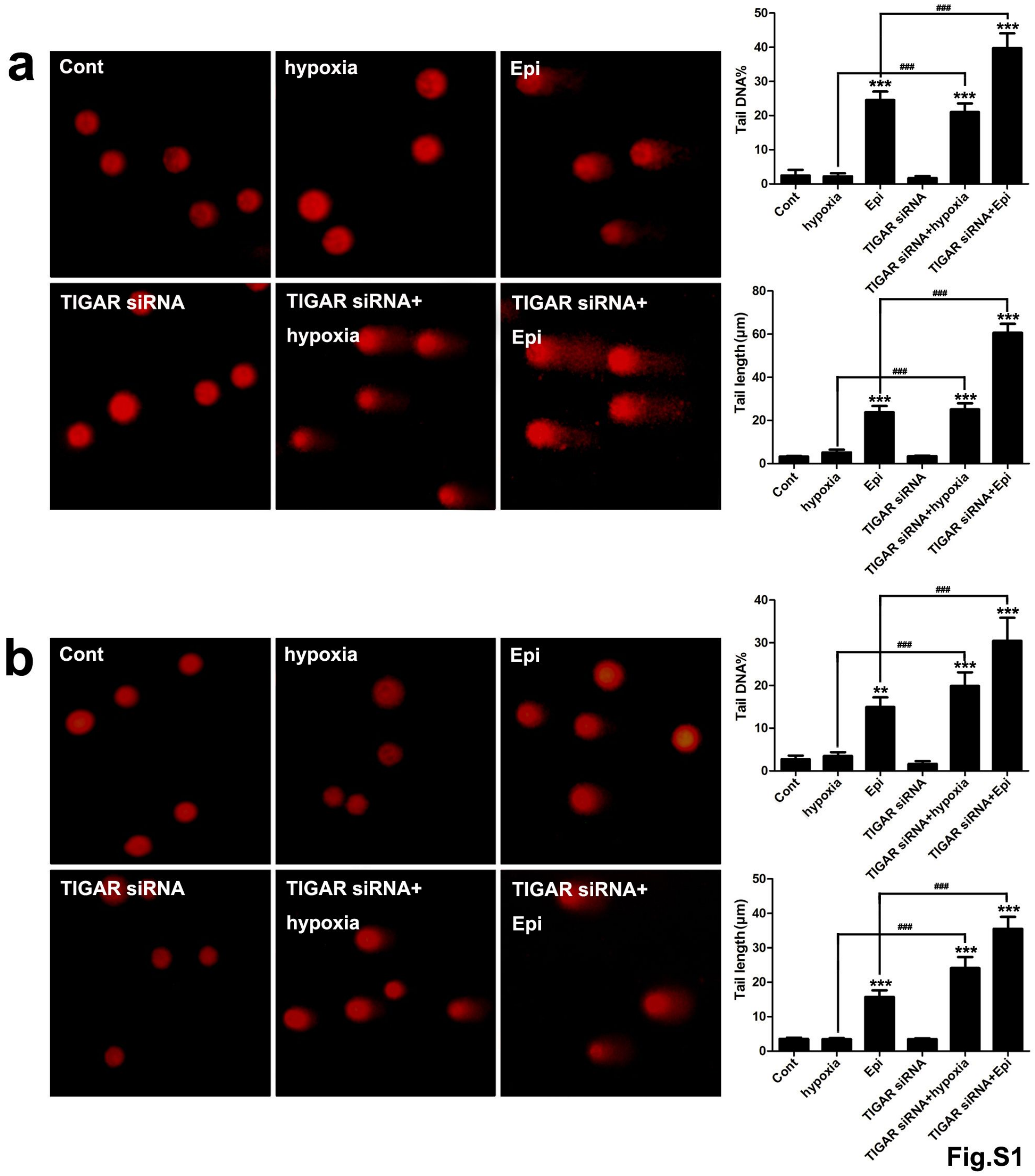
a
Cont
hypoxia
Epi
TIGAR siRNA
TIGAR siRNA+ hypoxia
TIGAR siRNA+ Epi
Tail DNA%
Tail length (μm)
Cont
hypoxia
Epi
TIGAR siRNA
TIGAR siRNA+hypoxia
TIGAR siRNA+Epi
***
###
b
Cont
hypoxia
Epi
TIGAR siRNA
TIGAR siRNA+ hypoxia
TIGAR siRNA+ Epi
Tail DNA%
Tail length (μm)
**
***
###

Fig.S1

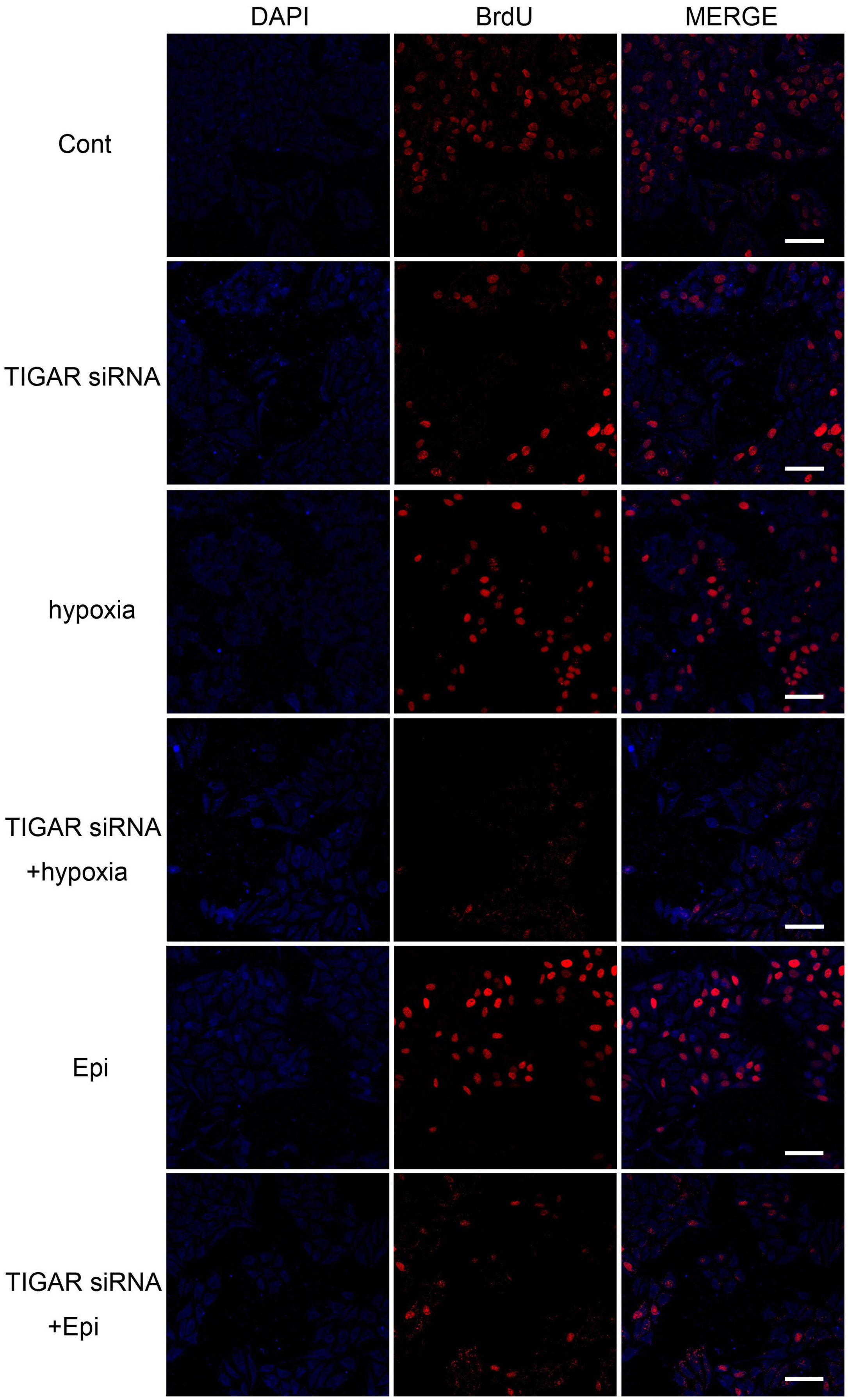
DAPI
BrdU
MERGE
Cont
TIGAR siRNA
hypoxia
TIGAR siRNA
+hypoxia
Epi
TIGAR siRNA
+Epi

Fig.S2

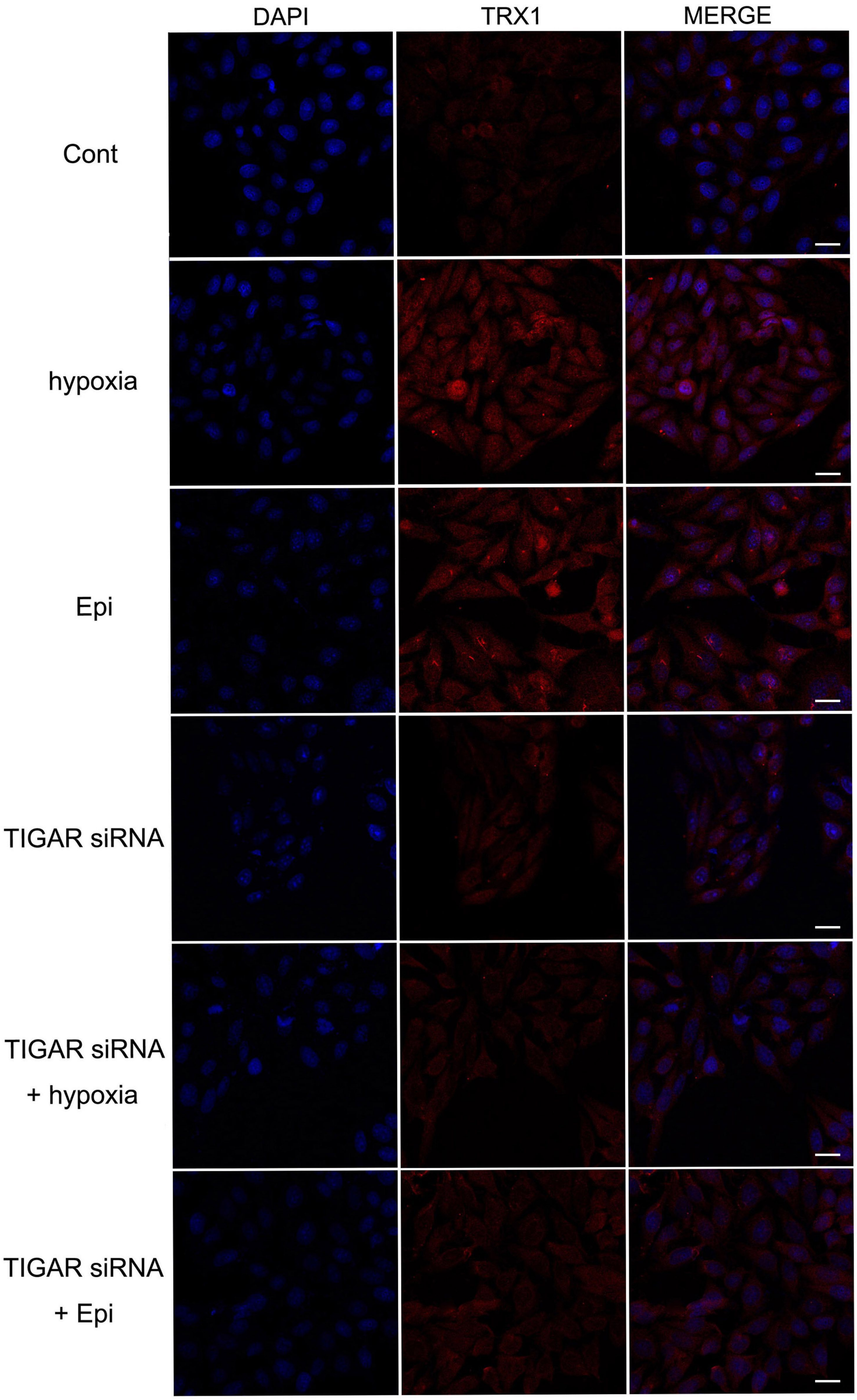
DAPI
TRX1
MERGE
Cont
hypoxia
Epi
TIGAR siRNA
TIGAR siRNA
+ hypoxia
TIGAR siRNA
+ Epi

Fig.S3

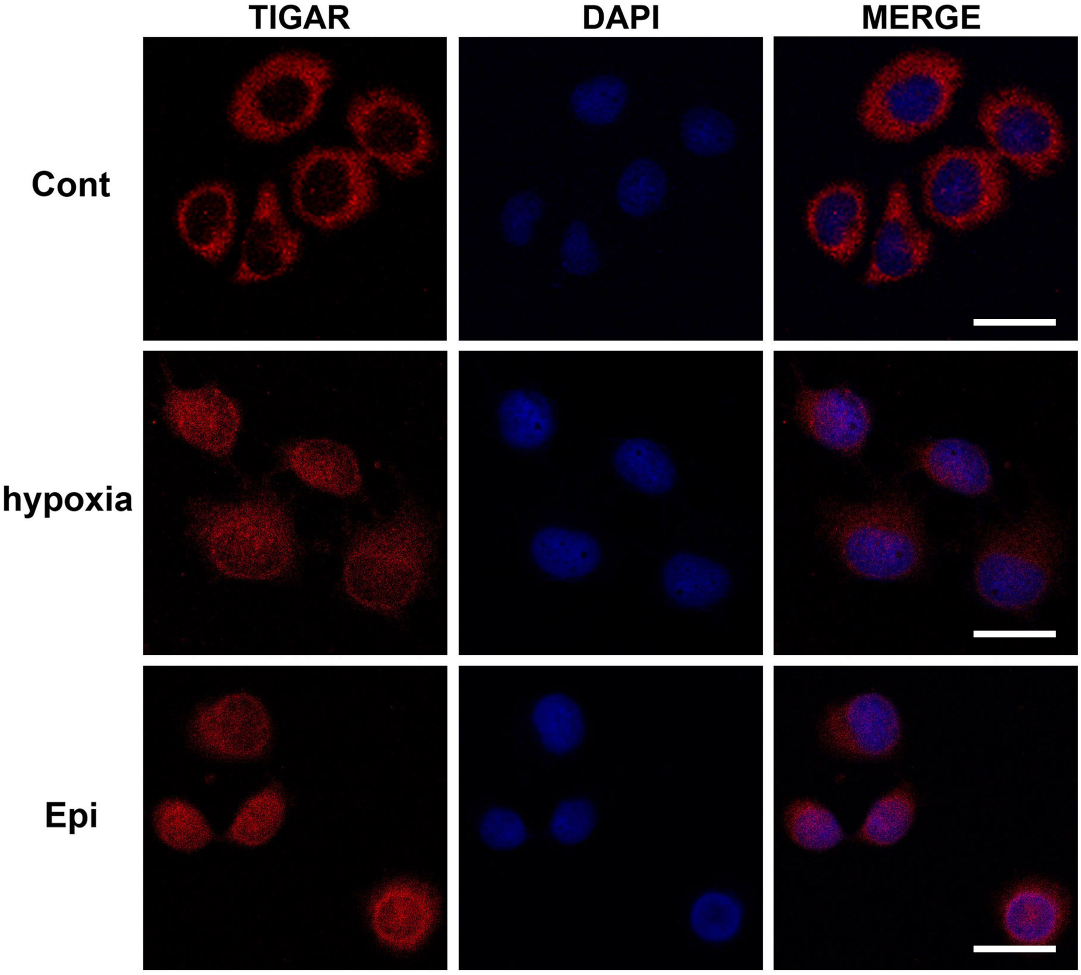

Fig.S4

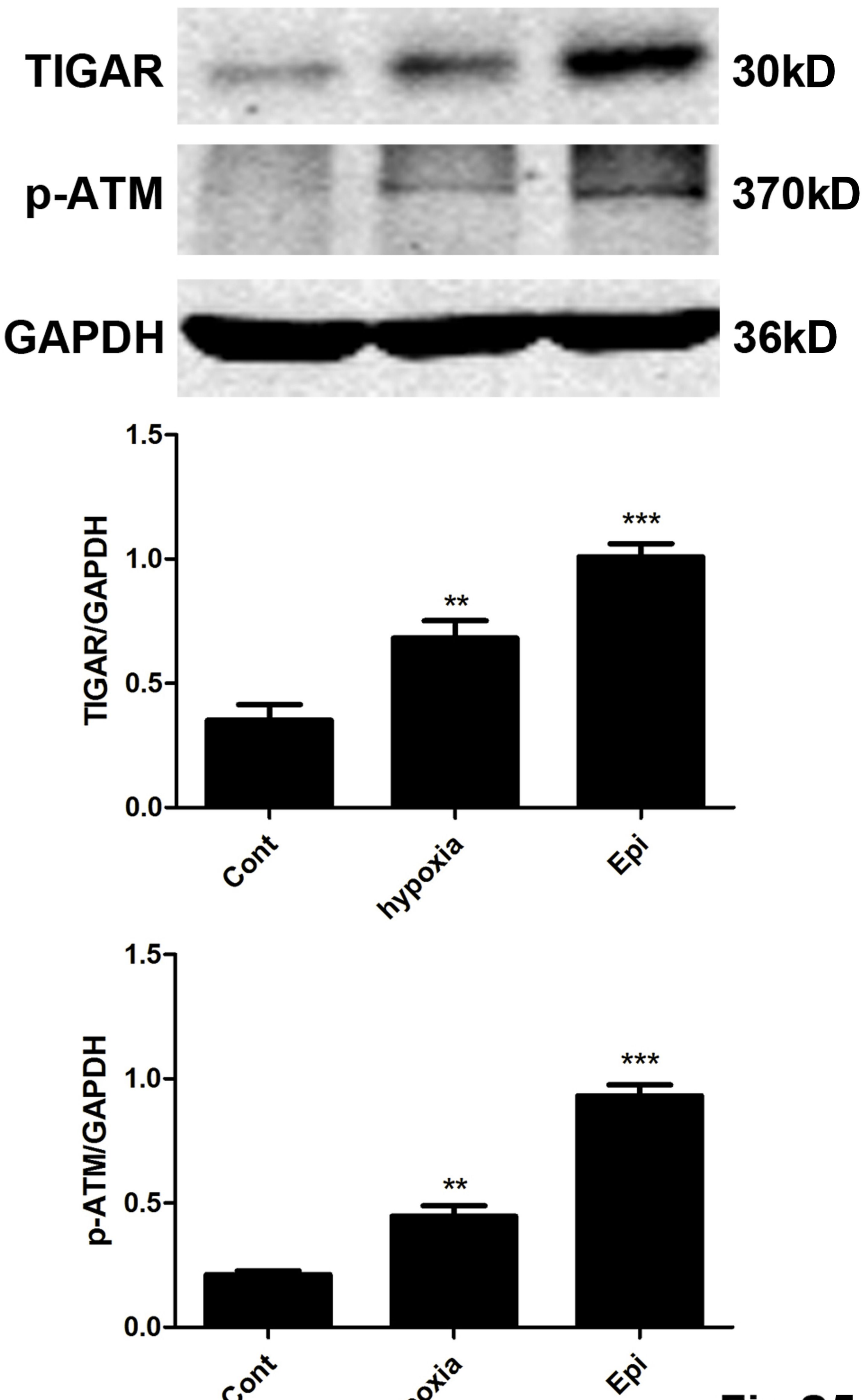
TIGAR
30kD
p-ATM
370kD
GAPDH
36kD
TIGAR/GAPDH
1.5
1.0
0.5
0.0
**
***
Cont
hypoxia
Epi
p-ATM/GAPDH
1.5
1.0
0.5
0.0
**
***
Cont
hypoxia
Epi

Fig.S5

Legends to figures:

Fig. S1 TIGAR knockdown increased DNA damage in H1299 and HTC116 cells. Knockdown of TIGAR in H1299 and HCT116 cells was achieved with transient transfection of TIGAR siRNA. Forty-eight h after transfection, H1299 and HCT116 cells were then treated with 200 mM $CoCl_2$ or 2.5 μg/ml epirubicin for 10 h and 12 h, respectively. (a) DNA damage caused by $CoCl_2$ or epirubicin in TIGAR knockdown H1299 cells. Left: representative images of Comet assay. Right: quantification of Comet tail DNA% and tail length. (b) DNA damage caused by $CoCl_2$ or epirubicin in TIGAR knockdown HCT116 cells. Left: representative images of Comet assay. Right: quantification of Comet tail DNA% and tail length. Values are means ± SD from 3 independent experiments. **p<0.01, *** p<0.001 versus control group; ### p<0.001 versus corresponding groups.

Fig. S2 TIGAR knockdown reduced DNA repair after epirubicin or CoCl2 treatment. HepG2 cells or TIGAR knockdown HepG2 cells were treated with 200 mM $CoCl_2$ or 2.5 μg/ml epirubicin. BrdU was added to culture medium 1 h before the end of experiment. Brdu positive cells were detected with a confocal microscopy. Brdu was stained red and the nucleus was stained blue. Scale bar =100 μm.

Fig. S3 TIGAR regulated the nuclear translocation of TRX1 in HepG2 cells. HepG2 cells or TIGAR knockdown HepG2 cells were treated with 200 mM $CoCl_2$ or 2.5 μg/ml epirubicin. The nuclear TRX1 protein was detected with a confocal

microscopy. TRX1 was stained red and the nucleus was stained blue. Scale bar = 25 μm.

Fig. S4 The nuclear translocation of TIGAR under genome stress or hypoxia condition in SMMC 7721 cells. The nucleus translocation of TIGAR after treatment of SMMC7721 cells with 200 mM $CoCl_2$ for 4 h or 2.5 μg/ml epirubicin for 12 h. The nuclear TIGAR was detected with a confocal microscopy. TIGAR was stained red and the nucleus was stained blue. Scale bar = 25 μm.

Fig. S5 TIGAR and ATM were induced by epirubicin or $CoCl_2$ in SMMC7721 cells. SMMC7721 cells were treated with 200 mM $CoCl_2$ for 8 h or 2.5 μg/ml epirubicin for 12 h. Expression of phosphorylated ATM and TIGAR proteins were detected with Western blot analysis. GAPDH was used as a loading control. Quantitative analysis was performed with Image J. Values are means ± SD from 3 independent experiments. **$p<0.01$, *** $p<0.001$ versus control group.

Full-length gels and blots

Figure 2 a

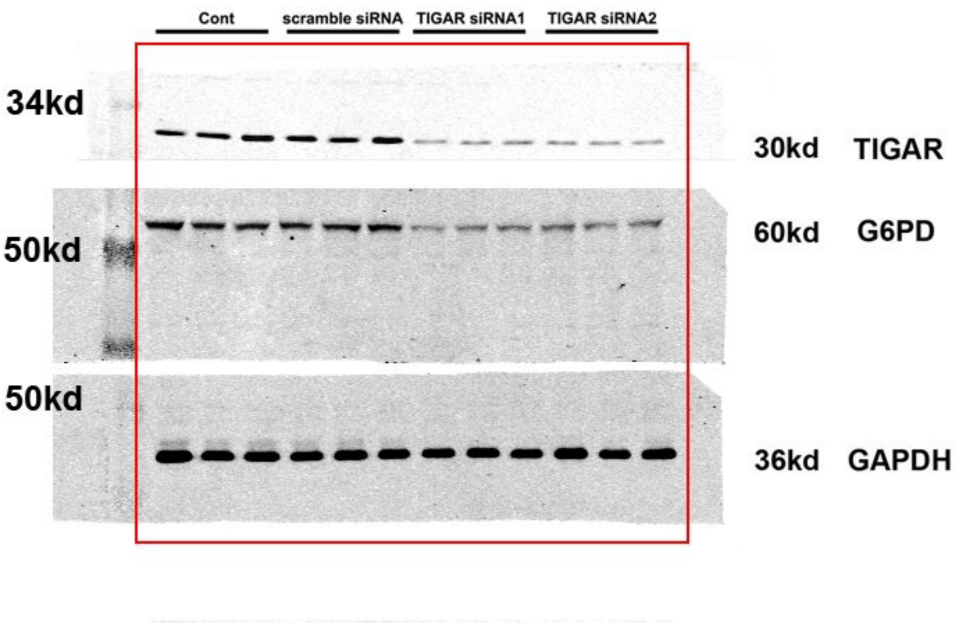

Figure 4 c

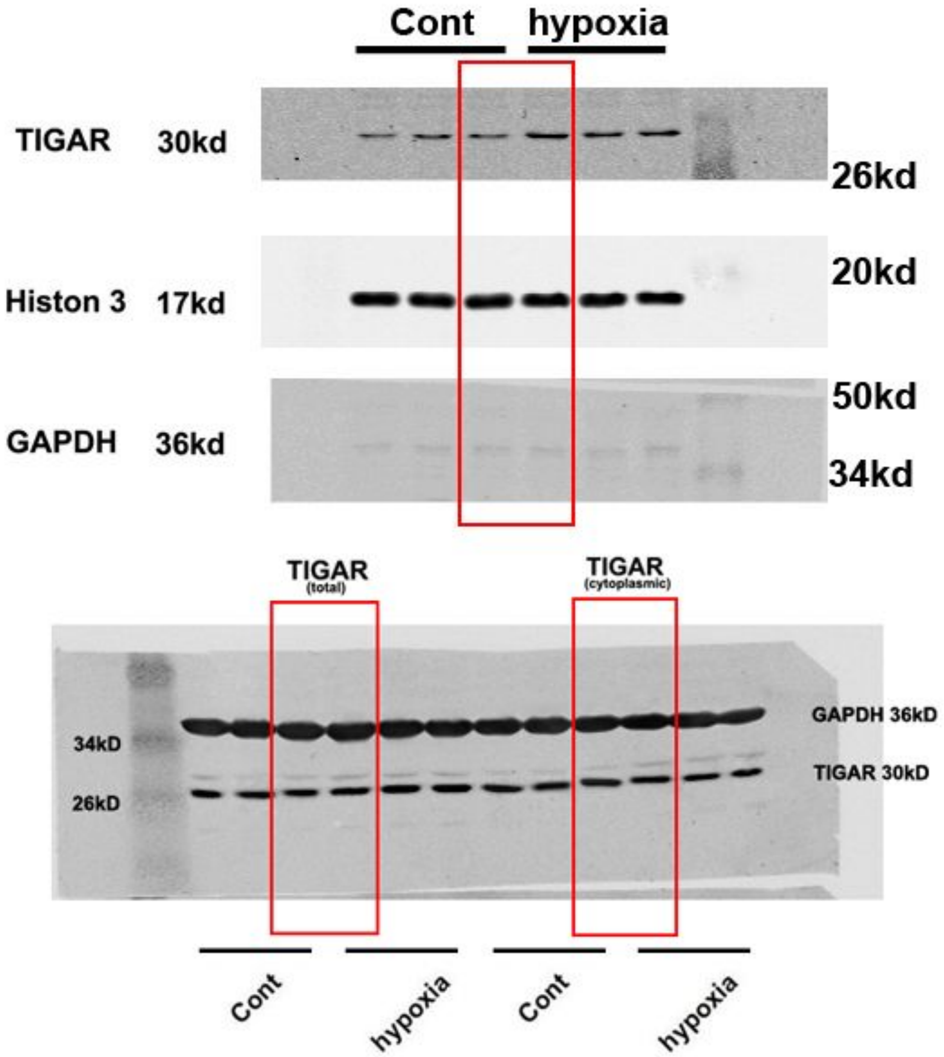
Cont
hypoxia
TIGAR
30kd
26kd
Histon 3
17kd
20kd
GAPDH
36kd
50kd
34kd
TIGAR
(total)
TIGAR
(cytoplasmic)
34kD
26kD
GAPDH 36kD
TIGAR 30kD
Cont
hypoxia
Cont
hypoxia

Figure 4 d

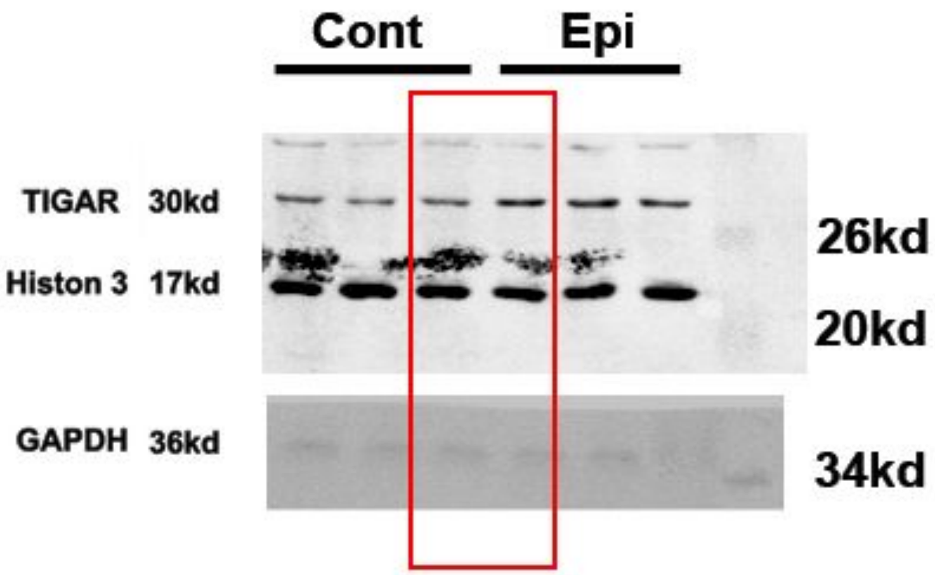

Cont
Epi
TIGAR 30kd
Histon 3 17kd
26kd
20kd
GAPDH 36kd
34kd

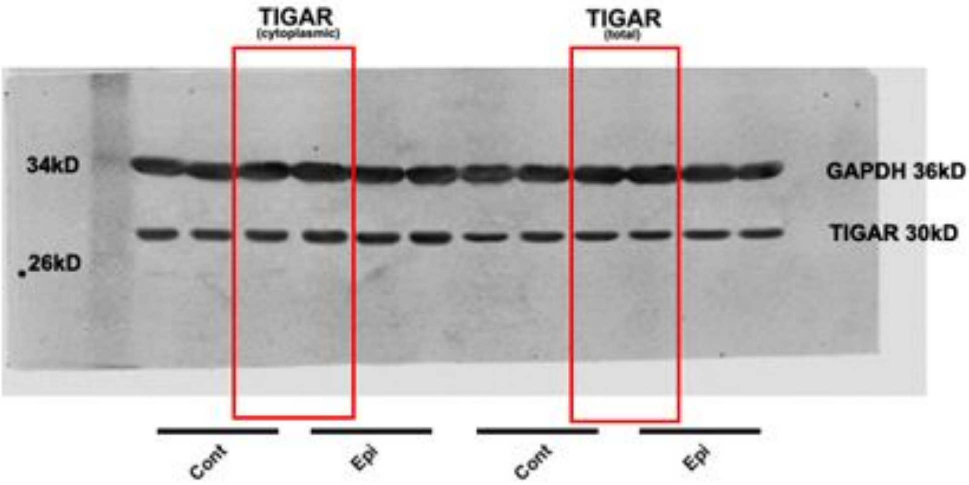

TIGAR
(cytoplasmic)
TIGAR
(total)
34kD
26kD
GAPDH 36kD
TIGAR 30kD
Cont
Epi
Cont
Epi

Figure 5 c

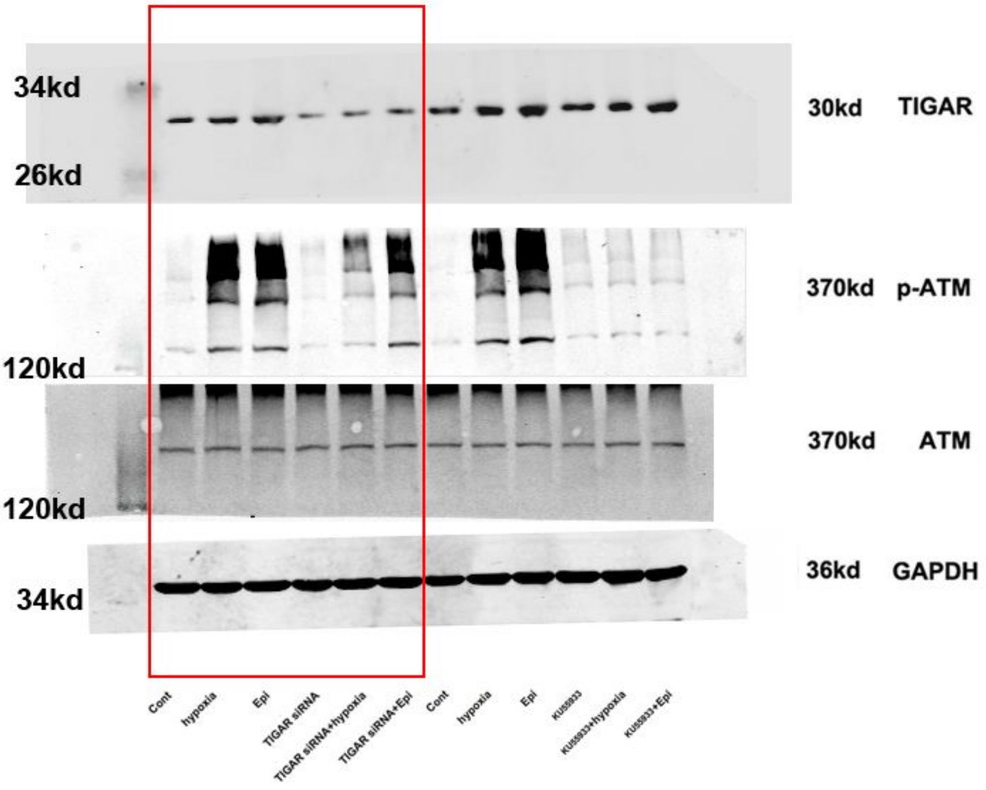
34kd
26kd
30kd TIGAR
370kd p-ATM
120kd
370kd ATM
120kd
36kd GAPDH
34kd
Cont
hypoxia
Epi
TIGAR siRNA
TIGAR siRNA+hypoxia
TIGAR siRNA+Epi
Cont
hypoxia
Epi
KU55933
KU55933+hypoxia
KU55933+Epi

Figure 5 d

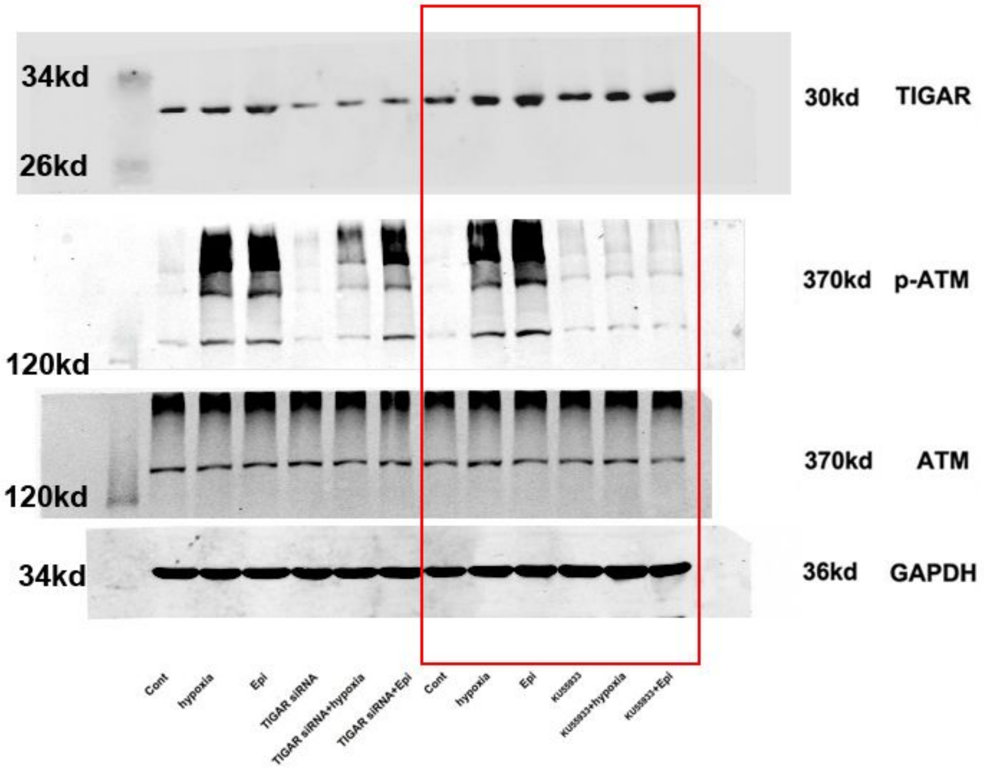

Figure 6 a

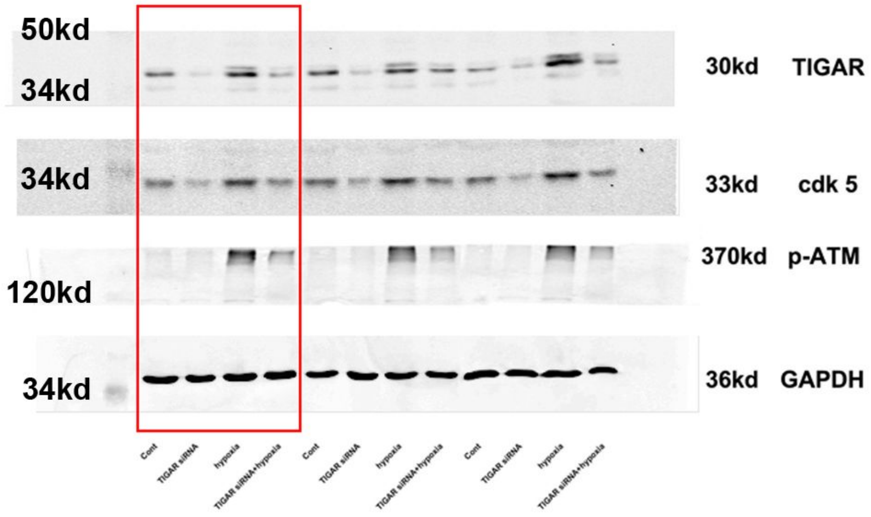

Figure 6 b

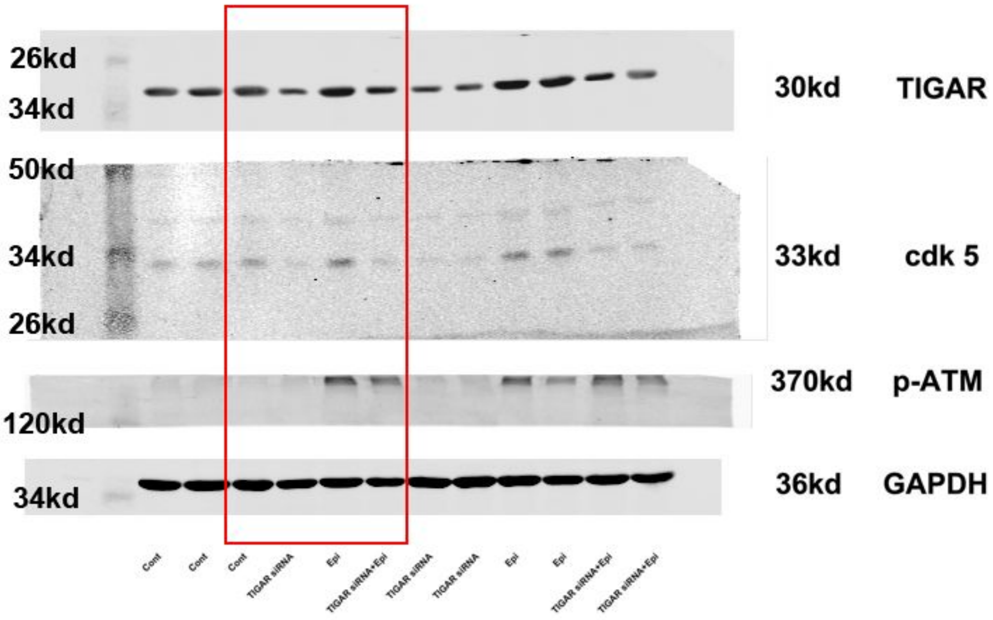

Figure 6 c

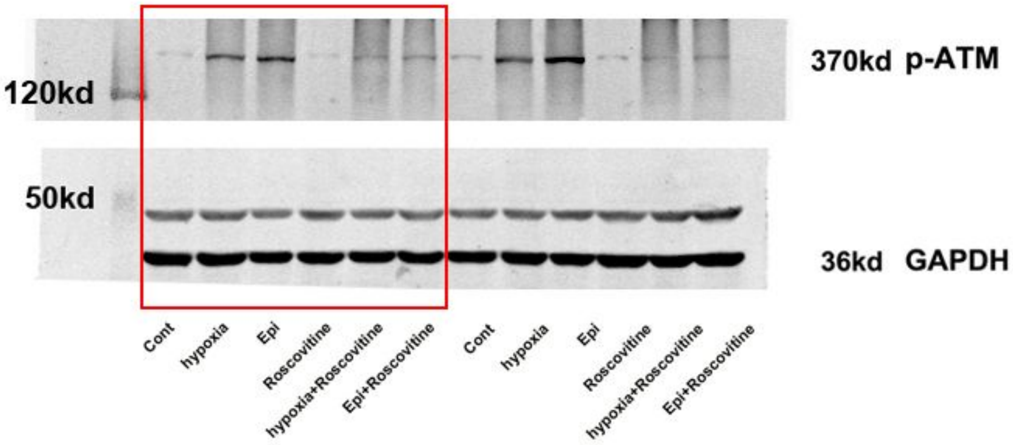

Figure S5

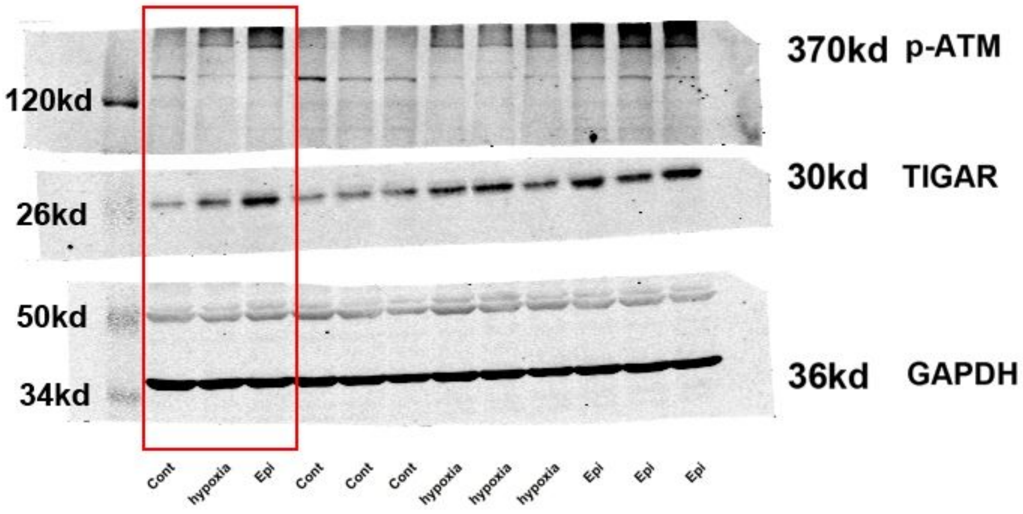

370kd p-ATM
120kd
30kd TIGAR
26kd
50kd
36kd GAPDH
34kd
Cont
hypoxia
Epi
Cont
Cont
Cont
hypoxia
hypoxia
hypoxia
Epi
Epi
Epi
